# Maternal obesity, length of gestation, risk of postdates pregnancy and spontaneous onset of labour at term

**DOI:** 10.1111/j.1471-0528.2008.01694.x

**Published:** 2008-05-01

**Authors:** FC Denison, J Price, C Graham, S Wild, WA Liston

**Affiliations:** a Division of Reproductive and Developmental Sciences, Centre for Reproductive Biology, Queen's Medical Research Institute Edinburgh, UK; b Department of Public Health Sciences, University of Edinburgh Edinburgh, UK; c Epidemiology and Statistics Core Unit, Wellcome Trust Clinical Research Facility, Western General Hospital Edinburgh, UK; d Department of Obstetrics and Gynaecology, Simpson Centre for Reproductive Health, Royal Infirmary Edinburgh, UK

**Keywords:** Body mass index, length of gestation, spontaneous onset labour

## Abstract

**Objective:**

To investigate the effect of maternal body mass index (BMI) on postdates pregnancy, length of gestation and likelihood of spontaneous onset of labour at term.

**Design:**

Retrospective cohort study.

**Setting:**

Swedish Medical Birth Register.

**Population:**

A total of 186 087 primiparous women (of whom 143 519 had spontaneous onset of labour at term) who gave birth between 1998 and 2002.

**Methods:**

Mann–Whitney test, one-way analysis of variance, linear regression and single variable logistic regression.

**Main outcome measures:**

Postdates pregnancy (≥294 days or 42^+0^ weeks), length of gestation and likelihood of spontaneous onset of labour at term.

**Results:**

About 6.8% of pregnancies delivered postdates. Higher maternal BMI (kg/m^2^) during the first trimester was associated with longer gestation (*P* < 0.001) as was a greater change in BMI between the first and third trimesters (BMI measured on admission prior to delivery) with mean (SD) gestation at delivery of 280.7 (8.6) and 283.2 (8.6) days for increases in BMI of <2 and ≥10 kg/m^2^, respectively. Higher BMI during the first trimester was associated with a lower chance of spontaneous onset of labour at term. Compared with BMI 20 to <25 kg/m^2^, the odds ratios (95% CI) for spontaneous onset of labour at term were 1.21 (1.15–1.27) for BMI of <20 kg/m^2^, 0.71 (0.69–0.74) for BMI of 25 to <30 kg/m^2^, 0.57 (0.54–0.60) for BMI of 30 to <35 kg/m^2^ and 0.43 (0.40–0.47) for BMI of ≥35 kg/m^2^. Higher BMI during the first trimester (BMI of ≥35 kg/m^2^ compared with BMI of 20 to <25 kg/m^2^) was also associated with an increased risk of complications including stillbirth (OR 3.90, 95% CI 2.44–6.22), gestational diabetes (OR 5.61, 95% CI 4.61–6.83) and caesarean section (OR 2.39; 95% CI 2.20–2.59).

**Conclusions:**

Higher maternal BMI in the first trimester and a greater change in BMI during pregnancy were associated with longer gestation and an increased risk of postdates pregnancy. Higher maternal BMI during the first trimester was also associated with decreased likelihood of spontaneous onset of labour at term and increased likelihood of complications.

*Please cite this paper as:* Denison F, Price J, Graham C, Wild S, Liston W. Maternal obesity, length of gestation, risk of postdates pregnancy and spontaneous onset of labour at term. BJOG 2008;115:720–725.

## Introduction

Approximately 60 000 women per annum in the UK will deliver postdates.^1^ Postdates pregnancies are associated with an increased risk of intrapartum and postpartum obstetric complications and higher perinatal morbidity and mortality rates.^2^–^5^ Moreover, compared with babies delivered at term, babies delivered postdates are more likely to be hospitalised during the first 3 years of life and are at greater risk of developing conditions including epilepsy, neurodevelopmental deviation and Asperger's syndrome in later life.^6^–^9^

In an attempt to reduce maternal and perinatal risks, in particular those of late stillbirth or neonatal death, labour is often induced in postdates pregnancies. However, the ‘cost of induction’ includes increased medical intervention such as higher caesarean section and operative vaginal delivery rates.^10^ While some hold that the induction process *per se* is responsible for the greater intervention, there is increasing evidence that women whose pregnancies last beyond term are at higher risk of intervention whether delivery is induced or not.^11^

Despite postdates pregnancy being common, little is known about its prenatal risk factors.^12^,^13^ Therefore, developing and appropriately targeting interventions to reduce the risk of postdates pregnancy is difficult. It has been proposed that maternal body mass index (BMI) and nutrition may be involved in the timing of the onset of labour, possible operating through endocrine mechanisms.^14^ Women who are underweight are more likely to deliver preterm than those of normal weight^15^ with a study from Israel demonstrating that nutritional restriction may initiate labour.^16^ If the converse is true, then the women who are obese may be less likely to have a spontaneous onset of labour at term and be at increased risk of having a postdates pregnancy. These women, already at high risk due to their obesity, would be more likely to have their labour induced with a potential further rise in surgical intervention, morbidity and mortality.

The primary aim of this study was to investigate the effect of maternal BMI on the risk of postdates pregnancy, length of gestation and likelihood of spontaneous onset of labour at term. The secondary objective was to investigate the effect of maternal BMI on the risk of antenatal problems and mode of delivery.

## Methods

An anonymous database of 186 087 primiparous women with a singleton pregnancy who gave birth between 1998 and 2002 was obtained from the Swedish Medical Birth Register. This Register, based on copies of standardised medical forms, is well characterised and validated and contains medical data on 98–99% of deliveries in Sweden.^17^,^18^ From the first antenatal visit, prospective information on all hospital births is gathered by the midwife and obstetrician. The maternal variables recorded include age, smoking status, antenatal care and diagnoses, intrapartum and delivery outcome, gestation in days, height and weight measured during the first trimester and on admission prior to delivery. Infant characteristics include sex, live/stillborn, head circumference, weight and length. This information is forwarded to the Swedish Medical Birth Register through copies of standardised, individual antenatal, obstetric and paediatric records.^19^

Maternal BMI (kg/m^2^) was calculated from maternal weight and height measurements taken during the first (10–12 weeks) and third trimesters (on admission prior to delivery) and was grouped into the following categories: underweight (<20 kg/m^2^), normal (20 to <25 kg/m^2^), overweight (25 to <30 kg/m^2^), obese (30 to <35 kg/m^2^) and severely obese (≥35 kg/m^2^).^20^ Change in maternal BMI was defined as the difference between BMIs measured in the first and third trimesters. The BMI groups were categorised to take into account an indefinite number of decimal places in the continuous measure of BMI, for example a BMI group was defined as 20 to <25 kg/m^2^ as opposed to 20–24.99 kg/m^2^. Pre-existing hypertension, pre-eclampsia, gestational diabetes and antepartum and postpartum haemorrhage were classified using the Tenth revision of the International Classification of Diseases (ICD-10) codings. These diagnoses are made by medical staff at delivery and then forwarded onto the Swedish Medical Birth Register.

The main outcome measures were postdates pregnancy (defined as a pregnancy lasting ≥294 days compared with term gestation of 260–293 days), length of gestation and onset of spontaneous labour at term (defined as spontaneous onset of labour after 260 days). In the Swedish Medical Birth Register, pregnancy duration is estimated using a number of variables: date of last menstrual period, estimated date of delivery (from second-trimester ultrasound scan) and pregnancy duration as stated in the paediatric record. To derive the ‘best possible’ estimate of pregnancy duration, 12 hierarchical rules are then applied.^17^ In more than 97% of cases, pregnancy duration is based on agreement between calculated pregnancy duration with that stated on the paediatric record.^17^ Secondary outcome variables included pregnancy-associated complications including gestational diabetes, pre-existing hypertension and stillbirth.

We applied strict *a priori* exclusion criteria. Records with one or more of: missing gestation data, gestation <260 or >308 days, multiple births, not live birth, weight at delivery ≤40 or ≥190 kg, birthweight of ≤1000 g, birth length of <36 or >60 cm and head circumference of <27 or >58 cm were excluded. The gestational parameters chosen defined the research question and study population. The other parameters defined biological plausibility. Records outwith these parameters were excluded (0.06% of the initial dataset of 186 087 pregnancies) as they were most likely caused by recording and/or transcription errors. In addition, to investigate the effect of maternal BMI on length of gestation and the risk of postdates pregnancy, we used linear regression with gestation as the outcome variable. Therefore, those records that mentioned nonspontaneous labour, induction and caesarean section before contractions were excluded from this analysis because there had been an intervention that prevented us from ascertaining the true length of gestation.

The chair of the Lothian Research Ethics Committee (LREC03) confirmed that ethical approval was not required for this study because the study was using a database that was anonymised at source.

### Statistical analysis

A Mann–Whitney test was used to test the statistical significance of differences in maternal BMI between women who had had a postdates delivery and those who had not had a postdates delivery. A one-way analysis of variance (ANOVA) was used to test for an association between length of gestation and change in BMI. Linear regression was performed to determine which factors were associated with length of gestation, while single variable logistic regression was used to examine the association between BMI category and binary variables such pre-existing hypertension.

## Results

The study sample used to investigate the effect of maternal BMI on length of gestation and risk of postdates pregnancy comprised 143 519 pregnancies that ended in spontaneous labour (77% of the initial dataset of 186 087 pregnancies). Maternal age, anthropometric and demographic characteristics of this sample are summarised in Table 1. The proportion of this sample that were underweight, normal, overweight, obese and severely obese was 12.7, 59.2, 21.0, 5.2 and 1.7%, respectively. Overall mean gestation at delivery in those women with a spontaneous onset of labour at term was 281.3 (SD 8.4) days with 6.8% of pregnancies delivering postdates. Median BMI (interquartile range [IQR]) measured during the first trimester was higher in those mothers delivering postdates (23.4, IQR 21.5–26.0) compared with those delivering at term (22.9, IQR 21.0–25.3; *P* < 0.0001 using a Mann–Whitney test).

**Table 1:** Descriptive data for maternal age, anthropometrics and demographics for the study sample used to investigate the effect of maternal BMI on length of gestation and risk of postdates pregnancy

Maternal variables (number of women)	
Age (143 519), mean (SD)	27.5 (4.8)
Height (132 174), mean (SD)	166.7 (6.3)
Weight (126 358), median (IQR)	64.0 (58.0–71.0)
BMI at booking (kg/m^2^) (122 699), median (IQR)	22.9 (21.1–25.4)
BMI at delivery (kg/m^2^) (46 761), median (IQR)	28.3 (26.0–31.2)
Family situation: other and single, *n* (%)	9003 (6.7)
Smoking habits in early pregnancy, *n* (%)	
Nonsmoker	143 866 (89.0)
>1 per day	14 761 (11.0)

Increasing maternal age (*P* < 0.001), height (*P* < 0.001) and having a family situation other than living with the father (single or other; *P* = 0.025) were also associated with longer gestation, whereas smoking (*P* < 0.001) and being non-European (compared with European; *P* < 0.001) were associated with shorter gestation. The number of previous miscarriages was initially included in the model. However, this did not show a statistically significant relationship with gestational days when other factors were taken into account, and this variable was therefore removed from the final model. Thus, the linear regression model produced was: gestation = 260.61 + age × 0.09 + height × 0.10 + smoke ×−0.34 + 0.29 (if single) + 0.39 (if not single and not living with father) − 0.62 (if non-European) + BMI × 0.12. Although all of these variables showed highly statistically significant associations with gestational age, effect sizes were small and accounted for very little of the overall variation in gestational length (overall model *r*^2^ = 0.01).

Using a one-way ANOVA, a greater increase in BMI between the first and third trimesters was associated with longer gestation, with mean (SD) gestations of 281.7 (8.4), 280.7 (8.6), 280.3 (8.4), 281.3 (8.3), 282.2 (8.1), 282.8 (8.3) and 283.2 (8.6) days for increases in BMI of 0, 0 to <2, 2 to <4, 4 to <6, 6 to <8, 8 to <10 and ≥10 kg/m^2^, respectively. Data for only 43 783 (30.5%) mothers were included in this analysis due to missing BMI data at time of delivery.

The study sample used to investigate the effect of maternal BMI on spontaneous onset of labour, stillbirth, maternal complications and caesarean section consisted of 173 174 pregnancies (93% of the initial dataset of 186 087). This sample differed from the previous sample in that those women with nonspontaneous labour, induction and caesarean section before contractions were included. The characteristics of this study sample are summarised in Table 2. The percentage of this sample that were underweight, normal, overweight, obese and severely obese was 12.1, 58.1, 21.9, 5.8 and 2.0%, respectively.

**Table 2:** Descriptive demographics and outcome data for the study sample used to investigate the effect of maternal BMI on likelihood of spontaneous onset of labour, maternal complications, stillbirth and caesarean section

Maternal variables (*n*)	
Age, mean (SD)	27.5 (4.8)
BMI at booking (kg/m^2^) (148 014), median (IQR)	23.1 (21.2–25.6)
Pre-existing hypertension, *n* (%)	214 (0.2)
Pregnancy-induced hypertension, *n* (%)	3371 (2.4)
Diabetes, *n* (%)	1033 (0.7)
Premature rupture of membranes, *n* (%)	413 (0.3)
Antepartum haemorrhage, *n* (%)	613 (0.4)
Postpartum haemorrhage, *n* (%)	7579 (5.3)
Caesarean section, *n* (%)	12 936 (9.0)

The higher the maternal BMI during the first trimester, the lower the likelihood of spontaneous onset of labour at term (Table 3). The risks of having pre-existing hypertension, a stillbirth or postpartum haemorrhage or developing gestational hypertension or gestational diabetes during pregnancy were also associated with higher maternal BMI in the first trimester. There was no association between maternal BMI and antepartum haemorrhage (Table 3). As first-trimester maternal BMI increased, so did the risk of having a caesarean section compared with any other type of delivery (normal, ventouse or forceps) during the third trimester. Women in the lowest BMI category (<20 kg/m^2^, conventionally considered underweight) were more likely to go into spontaneous labour and had a lower likelihood of various complications, including hypertension, gestational diabetes and caesarean section when compared with those with a normal BMI (Table 3).

**Table 3:** Odds ratios and 95% CIs tabulated for each variable according to BMI grouping at booking and in comparison with a normal BMI of 20 to <25 kg/m^2^

	BMI category* (kg/m^2^)				
	<20	20 to <25	25 to <30	30 to <35	≥35
Spontaneous onset of labour	1.21 (1.15–1.27)	1.00	0.71 (0.69–0.74)	0.57 (0.54–0.60)	0.43 (0.40–0.47)
Stillbirth	0.52 (0.31–3.87)	1.00	1.70 (1.31–2.22)	2.87 (2.03–4.04)	3.90 (2.44–6.22)
Pre-existing hypertension	0.54 (0.33–0.92)	1.00	2.30 (1.80–2.93)	4.82 (3.60–6.45)	9.07 (6.45–12.75)
Pregnancy-induced hypertension	0.71 (0.64–0.78)	1.00	1.80 (1.71–1.91)	2.90 (2.68–3.13)	4.24 (3.81–4.72)
Gestational diabetes	0.55 (0.44–0.70)	1.00	1.91 (1.69–2.15)	2.99 (2.54–3.52)	5.61 (4.61–6.83)
Antepartum haemorrhage	0.96 (0.79–1.16)	1.00	1.02 (0.88–1.19)	0.77 (0.58–1.04)	1.08 (0.71–1.65)
Postpartum haemorrhage	0.90 (0.83–0.97)	1.00	1.11 (1.05–1.18)	1.19 (1.08–1.31)	1.35 (1.16–1.56)
Caesarean section (yes/no)	0.74 (0.71–0.78)	1.00	1.45 (1.40–1.50)	1.87 (1.77–1.97)	2.39 (2.20–2.59)

* Baseline comparison with BMI of 20 to <25 kg/m^2^.

## Discussion and conclusion

We demonstrate that in pregnancies that extend beyond 260 days, a higher maternal BMI during the first trimester was associated with increased risk of postdates pregnancy. A greater increase in maternal BMI between first and third trimesters was also associated with longer gestation. In addition, maternal BMI in the first trimester influenced the risk of spontaneous onset of labour at term. The proportion of women with a BMI of 35 kg/m^2^ or more in the first trimester who went into spontaneous onset of labour at term was approximately 50% lower than those with a normal BMI in the first trimester.

Our findings are derived from analysing the variables contained within a pre-existing database. We therefore acknowledge that potential confounding factors not considered here, such as social class, family and medical history may have affected the relationship between maternal obesity and postdates pregnancy. In addition, the policy for management of postdates pregnancy may not be uniform throughout Sweden, and by using a national birth register, we may have included nonhomogeneous subjects treated according to different policies within the same cohort. Due to the anonymous nature of the database provided, we were not able to link individuals back to a particular hospital at delivery and therefore could not investigate this issue further. However, unless there was a systematic bias in the induction policies between different centres (e.g. dependent on maternal BMI at booking), this would be unlikely to affect our findings. In addition, we acknowledge that the proportion of women in this database who had a BMI of ≥30 kg/m^2^ was significantly lower than recent studies in UK antenatal populations.^21^,^22^ This may reflect the fact that the prevalence of obesity is lower in Sweden than in the UK and that the database used spanned the years 1998–2002 when overall rates of obesity were lower.^23^

An association between higher maternal BMI in early pregnancy and increased risk of postdates pregnancy is of clinical and public health importance. If the relationship proves causal, then we would expect an increase in the prevalence of postdates pregnancy due to the rising rate of maternal obesity nationally. The present findings from routinely collected data support the need for a prospective epidemiological study including a wide range of potential confounding factors to fully evaluate the risk factors for postdates pregnancy and the potentially causal contribution that maternal obesity makes to this risk.

The finding that a change in maternal BMI affects length of gestation has been reported previously.^24^,^25^ A case–control study (*n* = 3191) demonstrated that increased maternal gestational weight gain was associated with a higher risk of postterm delivery.^24^ This study was limited in that weight gain in pregnancy was calculated by subtracting a self-reported pre-pregnancy weight, determined retrospectively by maternal recall, from the last recorded maternal weight prior to delivery when the mean length of time between last prenatal visit and delivery was 6.1 ± 6 days. Both of these measurements may therefore have been subjected to recording bias. A second study also found that excessive gestational weight gain was associated with prolonged pregnancy.^25^ This study differed from the current study in that it was undertaken in a Chinese population and was limited by small numbers (76 overweight and 476 normal weight women). By using accurate methods of determining change in maternal BMI and gestation in a large database (43 783), we have shown that the greater the increase in maternal BMI during pregnancy, the longer the gestation. This relationship holds true, regardless of the starting BMI in early pregnancy. Our findings, however, differ from that of Olesen *et al.* (2006) who found that gestational weight gain per day of pregnancy did not influence risk of postterm delivery.^13^ This discrepancy might be due to selection bias in our study as only 30.5% of women had their BMI recorded during the third trimester on admission prior to delivery; potentially, women with extremes of BMI might have been more likely to have had their BMI recorded, thus introducing bias. Alternatively, this may be due to the greater size of our study, the way Olesen *et al.* considered weight gain in their study or the fact that only 35% of Danish women participated in the Danish Birth Cohort during their study period, thus potentially introducing bias into their study population.

The factors that control length of gestation and onset of parturition are not well understood. However, circulating levels of corticotrophin-releasing hormone, mainly synthesised by the placenta,^26^ and cortisol are significantly lower in maternal plasma at 22–24 weeks in women who deliver at term compared with those who deliver preterm.^27^ Moreover, longitudinal studies demonstrate a less rapid rise in maternal corticotrophin-releasing hormone in women who deliver postdates compared with those who deliver term or preterm.^28^,^29^ Although obesity is associated with activation of the hypothalamic–pituitary–adrenal axis, cortisol clearance is also increased and plasma cortisol levels are often low or normal.^30^–^32^ In addition, in nonpregnant women, there is a clear-cut inverse linear correlation between plasma cortisol level and relative weight.^33^ Obese women may therefore have lower circulating cortisol levels during pregnancy than those of normal weight. This could reduce placental corticotrophin-releasing hormone production and consequently influence timing of delivery.^34^ Alternatively, in obese women, the concentration of estrogen in adipose tissue may result in a reduction in levels of circulating estrogen and an alteration in the estrogen:progesterone ratio in maternal plasma, which increases prior to normal delivery.^35^ To date, no studies has investigated the effect of maternal obesity on uteroplacental biology, the hypothalamopituitary axis and hormone trajectories during normal pregnancy, and it is likely that the mechanisms by which obesity influences the parturition trigger will be complex and multifactorial.^35^

In the UK, obesity has reached epidemic levels with the prevalence in women of all ages being estimated as 23% (13% for 16–24 years, 18% for 25–34 years and 22.1% for 35–44 years).^9^ Maternal obesity poses a significant risk to maternal and fetal health during pregnancy, and our study confirms the findings of others that obesity is associated with significant complications including stillbirth, gestational diabetes, pregnancy-induced hypertension and caesarean section.^36^–^39^ Moreover, in the most recent Confidential Report into Maternal Mortality, this risk was emphasised by the fact that more than 50% of all women who died from *Direct* on *Indirect* causes were either overweight or obese and more than 15% of all women who died from *Direct* or *Indirect* causes were morbidly or supermorbidly obese.^40^ Obesity is now the most common clinical risk factor in obstetric practice and is escalating with the rise in obese teenagers reaching reproductive age.^41^ Many observational studies have demonstrated the risks that obesity poses on pregnancy outcome. However, there is currently a paucity of interventional studies on obese women who have attempted to modify risk and improve pregnancy outcome. Despite this, it is now very clear that a pregnancy in an obese woman should be considered as high risk. Moreover, antenatal care should be individualised in obese women and delivered by a multidisciplinary team to reduce risk and improve maternal and fetal outcome. Finally, being very slim with a BMI of <20 kg/m^2^ also confers some benefits, in terms of increased probability of spontaneous labour and reduced risk of hypertension, diabetes and caesarean section compared with those with a BMI of 20 to <25 kg/m^2^. Potentially, if a healthy lifestyle including physical activity and healthy eating, which are more common in underweight women, were advocated more strongly for the obese obstetric population, then obstetric outcomes might be improved.

## Funding

This study was funded by TENOVUS, Scotland. All the authors state their independence from the funders.

## Contribution to authorship

F.D. initiated the design of the study with W.A.L. and was involved with the development and conduct of the study, coordinated analyses, wrote drafts of the paper and reviewed the paper. She is guarantor. J.P. and S.W. reviewed analyses, interpreted results and participated in writing and reviewing of the paper. C.G. had the major role in data management and carried out the statistical analyses. W.A.L. initiated the study design with F.D., interpreted results and participated in the writing and reviewing the paper.
